# The Free Flap Reconstruction of Facial Defects after Squamous Cell Carcinoma Excision

**DOI:** 10.3390/medicina60091432

**Published:** 2024-09-02

**Authors:** Tae-Yul Lee, Seungjun Lee, Seokchan Eun

**Affiliations:** 1 Department of Plastic and Reconstructive Surgery, Korea University Ansan Hospital, Ansan 15355, Republic of Korea; tylee0919@korea.ac.kr; 2 Institute of Advanced Regeneration and Reconstruction, Seoul 02841, Republic of Korea; 3 Department of Plastic and Reconstructive Surgery, Seoul National University College of Medicine, Seoul National University Bundang Hospital, Seongnam 13620, Republic of Korea

**Keywords:** squamous cell carcinoma, facial reconstruction, free flap, outcomes

## Abstract

*Background and Objectives:* Cutaneous squamous cell carcinoma is the second most common skin cancer. There are many methods for the reconstruction of facial subunit defects after skin cancer excision. The face is vital to a person’s life and should be reconstructed considering functional and aesthetic aspects. Despite a variety of flap types and techniques, it is still challenging to meet the various demands. The aim of this study was to compare free flaps for facial reconstruction after resection of cutaneous squamous cell carcinoma. *Materials and Methods:* This study included 14 patients from January 2021 to June 2023. Patients who underwent facial SCC resection and subsequent reconstruction using free flaps were analyzed retrospectively. Age, sex, and localization were recorded. Follow-ups ranged from 5 to 21 months, with an average of 13 months. *Results:* All free flaps survived well except one case of partial flap necrosis. In most patients, good to excellent functional and aesthetic results were obtained. The donor site healed uneventfully in all patients. *Conclusions:* Free flap reconstruction is an excellent choice in wide skin oncologic defects. In terms of texture, it also could be a good surgical method. The use of a fraxel laser can progressively facilitate improved color matching with the surrounding skin.

## 1. Introduction

Facial squamous cell carcinoma (SCC) represents a prevalent type of skin cancer characterized by the malignant proliferation of keratinizing cells within the epidermis [1,2]. Despite the availability of various treatments for skin cancer, surgery remains the cornerstone for cutaneous squamous cell carcinoma, emphasizing complete surgical excision with histopathologically clear resection margins as the gold standard [3,4]. While several local flaps are adept at covering facial defects, their utility is often constrained to smaller and medium-sized defects due to inherent limitations [5,6,7]. Skin cancer ablative surgery can lead to substantial soft tissue defects, necessitating intricate reconstruction. The face, segmented into distinct functional and aesthetic subunits, presents unique challenges, making the restoration of each subunit particularly demanding. Microvascular flaps emerge as a viable option for larger defects, despite potential drawbacks such as poor color match and bulky tissue, a facet that has been underexplored in the existing literature [8]. This study aims to comprehensively evaluate various free flap techniques for oncologic defects post facial SCC resection and contribute valuable insights to the existing literature for future studies.

## 2. Methods

Between January 2021 and June 2023, our institution treated 14 patients with diverse facial defects resulting from squamous cell carcinoma resection. The treatment protocol involved immediate microsurgical reconstruction utilizing various free flap methods, taking into account factors such as defect size, depth, location, recipient vessels, and required tissue composition. The patient cohort, comprising 8 males and 6 females with an average age of 66.7 years (range: 49–89 years), presented lesions in the temple, lower eyelid, cheek, ala, preauricular region, lower lip, and chin. A comprehensive retrospective review of patient records, radiographs, and photographs provided essential data on tumor size, location, defect size post-resection, safety margin, flap type and size, and recipient pedicles. Complications, including recurrence, infection, wound dehiscence, and partial or total flap loss, were meticulously investigated. This study obtained approval from the institutional review board of the Seoul National University Bundang Hospital (B-2105-685-101). Furthermore, this study obtained the patients’ informed consent by thoroughly explaining the procedure and obtaining their signature.

The criteria for selecting the type of reconstruction were as follows: the location of the patient’s SCC and the tumor size were considered to predict the potential defect size and the required pedicle length. If a large defect was anticipated, the anterolateral thigh flap was primarily considered. For cases requiring thin tissue and a long pedicle, the radial forearm free flap was selected, whereas for smaller defects requiring a short pedicle, the posterior auricular free flap was preferred. In cases where a long pedicle was not necessary, the lateral arm free flap or the superficial circumflex iliac perforator flap was considered. All surgeries were performed under general anesthesia to ensure procedural stability. The method of postoperative follow-up involved monitoring the patient’s condition to ensure stable recovery, and it was conducted for at least one year. Free tissue transfer can result in a distinct scar with color inconsistency or hypertrophy immediately after surgery. To address this, we employed a widely used fraxel laser, performing serial resurfacing multiple times at 1- to 2-month intervals, starting from the flap margin and progressing towards the center.

## 3. Results

**Figure 1:**
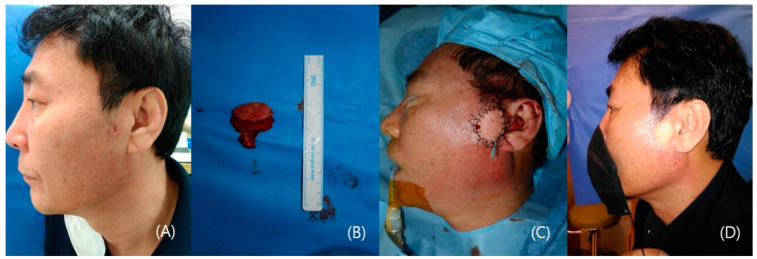
A case of squamous cell carcinoma on his left sideburn. (**A**) Pre-operative photo; (**B**) 3.2 × 2.5 cm lateral arm free flap harvested. (**C**) Immediate postoperative photo; (**D**) 4-month postoperative photo.

**Figure 2:**
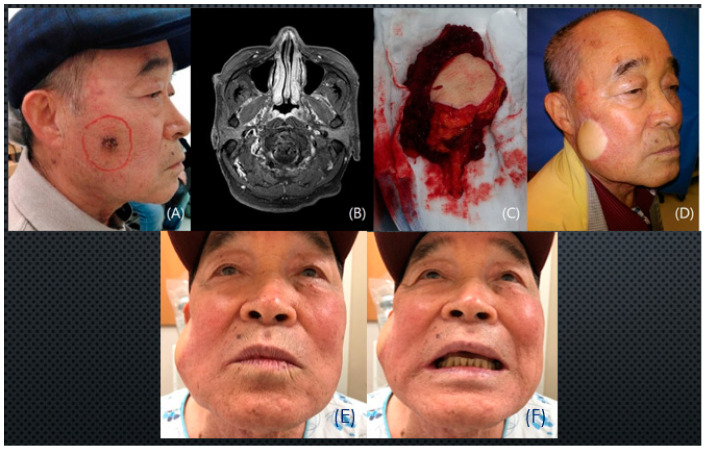
A case of a cheek squamous cell carcinoma patient. (**A**) Pre-operative photo. (**B**) Pre-operative MRI finding of facial nerve involvement; (**C**) 5 × 5 cm anterolateral thigh chimeric musculocutaneous flap for facial reanimation; (**D**) 11-month postoperative photo; (**E**,**F**) 11-month postoperative photo; the frontal view shows dynamic functional restoration.

All soft tissue defects resulting from tumor resection were successfully reconstructed, highlighting the efficacy of the employed microsurgical techniques. The average operation time, a critical metric in evaluating procedural efficiency, was recorded at 190 min (range: 163–251 min). The subsequent healing time at the surgical site averaged 11 days (range: 5–19 days), underscoring the prompt recovery observed in our patient cohort. Histological examination affirmed the complete removal of all SCCs, with both margins and the lesion’s base demonstrating negative results. The reconstruction of 14 facial defects entailed the use of 14 distinct free flaps, including 3 anterolateral thigh flaps, 3 radial forearm flaps, 3 superficial circumflex iliac artery perforator flaps, 4 lateral arm flaps, and 1 preauricular flap (refer to Table 1 for detailed information). It is noteworthy that one patient exhibited partial necrosis attributed to congestion after free flap reconstruction, a complication that successfully resolved through secondary intention. Throughout an average 13-month (range: 5–21 months) follow-up period, no abnormal findings or recurrences were observed in the physical examination of the reconstructed site and surrounding tissues. Moreover, the flaps exhibited a progressively less bulky appearance. Although some cases (Figure 1 and Figure 2) showed suboptimal color matching with the surrounding skin, the application of fraxel laser therapy led to observable transformations in the flaps, gradually achieving excellent outcomes with improved color assimilation to the adjacent skin. Encouragingly, no patients reported dissatisfaction with the aesthetic outcome post free flap surgery.

### 3.1. Case Series

#### 3.1.1. Case 1

In this case, a 60-year-old male with squamous cell carcinoma on the left sideburn underwent wide excision, resulting in a 3.2 × 2.5 cm defect. A lateral arm free flap was meticulously harvested and transplanted, with an end-to-end anastomosis to the preauricular branch of superficial temporal vessels. The procedure successfully restored facial functionality and aesthetics, allowing the patient to confidently expose his sideburn in daily life. This highlights the efficacy of lateral arm free flap reconstruction, emphasizing meticulous surgical technique for functional and aesthetic success post-squamous cell carcinoma ablation (Figure 1).

#### 3.1.2. Case 2

This report outlines successful facial reanimation in a 70-year-old male with a 1.5 × 1.2 cm squamous cell carcinoma on his right cheek, requiring extensive tumor ablation, including the zygomaticus major muscle. Immediate reconstruction involved an anterolateral thigh free flap, incorporating the vastus lateralis muscle and lateral femoral cutaneous nerve. The harvested flap was adeptly inserted to restore facial movement, and anastomosis of the lateral femoral cutaneous nerve connected the proximal facial nerve trunk to the zygomatic branch. A remarkable eleven-month postoperative follow-up revealed observable improvement in the lateral commissure movement, emphasizing the success of this reconstructive approach in achieving functional and aesthetic restoration (Figure 2).

#### 3.1.3. Case 3

**Figure 3:**
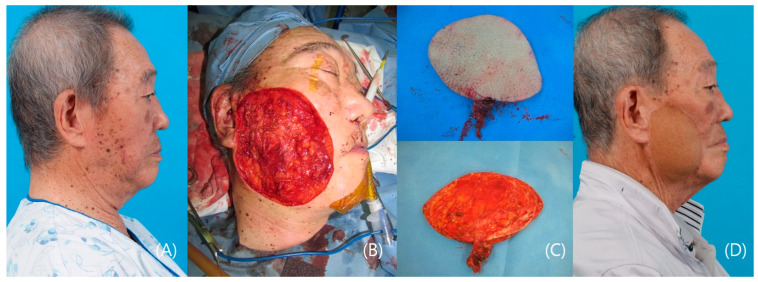
A case of right cheek squamous cell carcinoma patient. (**A**) Pre-operative photo; (**B**) 10 × 8 cm defect after wide excision; (**C**) 10 × 8 cm suprafascial anterolateral thigh flap. (**D**) Postoperative 1-year photo.

This patient is an 89-year-old male with squamous cell carcinoma. Ablation necessitated a superficial parotidectomy, revealing a substantial 10 × 8 cm sized extensive skin and soft tissue defect on the right cheek. An anterolateral thigh free flap was meticulously raised in the suprafacial layer, providing ample tissue coverage with minimal complications. The right facial artery and vein were used as recipient vessels. Postoperatively, reconstruction was achieved without complications, emphasizing the success of the procedure. The choice of the anterolateral thigh free flap and suprafacial layer contributed to oncologic well-being and a satisfying aesthetic outcome. The patient’s retained smiling motion highlighted the success of the reconstruction, emphasizing meticulous surgical planning and technique (Figure 3).

#### 3.1.4. Case 4

**Figure 4:**
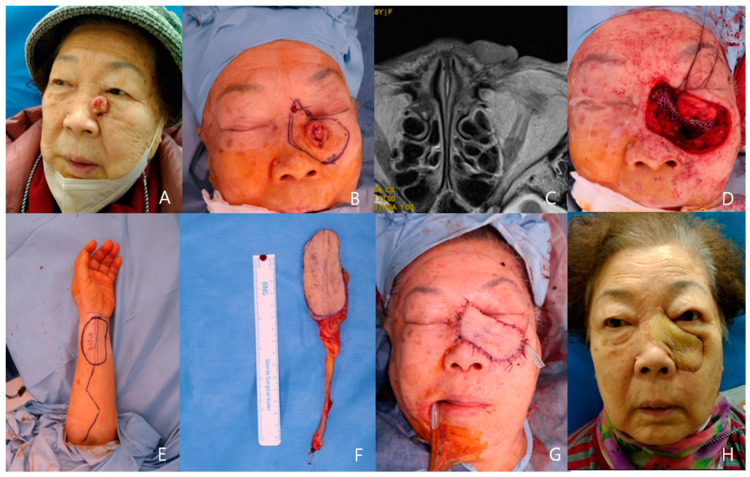
A case of left nasal wall and lower lid squamous cell carcinoma patient. (**A**) Pre-operative photo. (**B**) 2cm surgical safety margin. (**C**) MRI finding: 2 × 2 cm size and 8 mm depth SCC adjacent to bony wall. (**D**) After En-bloc excision of cancer and bony frame reconstruction using Synpor. (**E**) Radial forearm flap design. (**F**) Harvested flap. (**G**) Immediate postoperative view. (**H**) Post-operative 3 month view.

An 87-year-old female patient presented with squamous cell carcinoma on her left lower lid and lateral nasal wall area. Pre-operative MRI shows a 2 cm diameter protruding polypoid enhancing mass adjacent to the nasal bone and infraorbital rim. En bloc excision was performed including skin, subcutaneous tissue, the left nasal bone, the infraorbital rim, and part of the maxilla anterior wall. Bony framework was reconstructed with SYNPOR^®^ (Porous Polyethylene Implants, DePuy Synthes, New Brunswick, NJ, USA). A radial forearm free flap, 8 × 5 cm sized, was harvested and transferred to the facial defect area. The left facial artery and vein were used as recipient vessels. Postoperatively, successful reconstruction was achieved without complications (Figure 4).

## 4. Discussion

Cutaneous squamous cell carcinoma (cSCC) ranks as the second most prevalent skin cancer, necessitating facial reconstruction that meticulously considers both functional and aesthetic aspects [9]. The intricate nature of facial subunits, each characterized by unique skin thicknesses and tissue characteristics, underscores the challenges in achieving optimal postoperative outcomes. Local flaps, offering a color and texture match, are often limited in cases of extensive resection. Distant flaps introduce mismatches, and skin grafts, due to potential complications like contractions, hyperpigmentation, and heterogeneity, are often unsuitable. In this context, free flaps, despite potential inconsistencies in color, texture, and thickness, emerge as the preferred choice for large soft-tissue defects, offering versatility and reliable outcomes [10,11]. Wide excision coupled with free flap reconstruction has evolved into the mainstay of our skin cancer surgery. In the context of facial skin cancer, surgical resection serves as the primary treatment, with radical resection facilitated by the availability of free flaps. The classification and staging of cSCC, based on the TNM system, underscore the necessity of achieving two major surgical goals: complete removal of all cancer cells and aesthetically reasonable reconstruction [12,13,14]. The absence of incomplete resection, recurrence, or metastasis during our follow-up period further supports the efficacy of our chosen approach. Moreover, the individualization of safety margins based on tumor size, location, and stage demonstrates our commitment to achieving optimal oncologic outcomes while minimizing unnecessary tissue excision. This nuanced approach, particularly setting a 10 mm safety margin for all stage II cases, reflects our dedication to preventing local recurrence without compromising cosmetic results. We primarily reference the NCCN guidelines for margin assessment. For SCCs measuring 1 cm or larger and those located in high-risk areas (scalp, ears, eyelids, nose, lips), we apply a safety margin of at least 10 mm.

**Figure 5:**
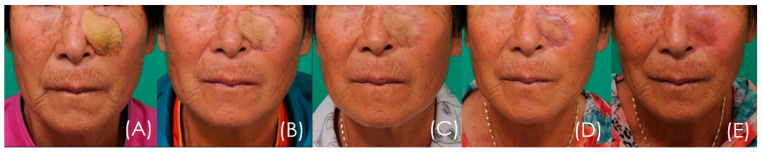
A left cheek squamous cell carcinoma patient demonstrates a sequential skin color change evaluation. (**A**) Postoperative 2-month view. (**B**) Postoperative 5-month view. First fraxel laser treatment applied to the flap margin. (**C**) Postoperative 8-month view. Second fraxel laser treatment applied to approximately half of the flap. (**D**) Postoperative 11-month view. Third fraxel laser treatment applied to the entire flap. (**E**) Postoperative 15-month view.

Our study contributes significantly to the understanding of postoperative outcomes. The observed tissue changes over time in terms of color and texture in all flaps signify an adaptive response that aligns with the surrounding skin (Figure 5). In particular, repeated fraxel laser treatments can aid the adaptive response. Figure 5 adequately illustrates the sequential changes in skin color. This evolution to a less-bulky appearance over time is an encouraging aspect of postoperative recovery that merits consideration in the broader context of reconstructive surgery. This study highlights not only the immediate success of free flap reconstruction but also the dynamic nature of tissue adaptation, an aspect that has been underexplored in the existing literature. While postoperative total flap loss remains a significant concern, the high success rate of free tissue transfer, ranging from 91% to 99%, is consistent with our findings. Our study recorded no instances of total flap necrosis, a testament to the reliability of the employed microsurgical techniques. However, the occurrence of partial flap necrosis in one patient emphasizes the importance of addressing unfavorable wound closure tension. This complication, though not unique to free flaps, warrants further investigation into optimal tension management strategies to enhance overall success rates.

Explaining the postoperative function of the flap can be somewhat challenging. Facial function cannot be solely assessed by facial movement, although it is undoubtedly an important aspect. Although dynamic functional restoration can be demonstrated through facial expressions, as shown in Figure 2E,F, it is also important to consider the static functional aspects. The primary functional requirement for a free flap covering defects resulting from skin cancer removal is to harmonize with other aesthetic units and support the function of the remaining normal tissue. For instance, in Figure 3D, the right mouth corner maintains its pre-operative level without drooping, demonstrating adequate functionality. Controlling facial movement with an ALT flap, which does not include muscle, is impossible. Another example, as shown in Figure 4H, involves primary skin cancer that is very close to the left lower lid with bony frame involvement. In this case, severe ectropion, nasal contour deformity, or nasal airway collapse could have occurred. However, these complications did not arise following reconstruction with a radial forearm free flap, thereby preserving functional integrity.

Each free flap possesses distinct advantages and disadvantages. Therefore, rather than having absolute contraindications, the concept is to understand the characteristics of each free flap thoroughly and apply them according to the specific features of the defect. For instance, the suprafascial ALT flap can provide a thin flap and a long pedicle, making it a viable alternative to the RFFF. Similarly, the SCIP flap, with its relatively shorter pedicle, can be used in place of the LAFF.

This study has several limitations. First, given that the sample size is limited to 14 patients, no special statistical data processing methods were employed. However, as more patient data are accumulated in the future, we plan to conduct additional research utilizing statistical methods to analyze data related to surgical interventions. Second, the scope of our study is limited to patients who underwent surgery between January 2021 and June 2023, thus restricting detailed discussion on long-term evolution. Third, this study is unfortunately limited by the insufficient availability of follow-up photographs. In some cases, it is difficult to compare immediate postoperative photos with long-term follow-up images. Lastly, our study is a retrospective study; it is not feasible to conduct an analysis comparing free flaps with other reconstruction methods.

## 5. Conclusions

In conclusion, our study not only provides a comprehensive evaluation of various free flap techniques for oncologic defects post facial SCC resection but also sheds light on the dynamic nature of tissue adaptation over time. The use of fraxel laser can progressively facilitate improved color matching with the surrounding skin. While acknowledging the potential for complications such as partial flap necrosis, our results underscore the overall success and reliability of free flap reconstruction in addressing facial defects resulting from squamous cell carcinoma resection. Wide excision coupled with free flap reconstruction emerges as a robust approach, ensuring both the removal of cancer cells and the achievement of aesthetically reasonable reconstruction. The individualization of safety margins based on tumor characteristics further enhances the precision of our surgical approach, emphasizing our commitment to achieving optimal outcomes in both oncologic and aesthetic dimensions.

## Figures and Tables

**Table 1:** Patients’ demographics.

N	Sex	Age	Site	Safety Margin	Tumor Size	Flap	Flap Size	Complications
1	M	65	Lower eyelid	20 mm	2 × 1 cm	RFFF	6 × 4 cm	Mild lid-lag
2	M	67	Cheek	10 mm	2 × 1 cm	SCIP	4 × 3 cm	Partial necrosis
3	F	81	Temple	10 mm	2.2 × 1.3 cm	LAFF	5 × 3 cm	None
4	M	55	Ala	6 mm	1 × 1 cm	PAFF	3 × 3 cm	Mild bulkiness
5	M	60	Preauricular	10 mm	1.2 × 0.5 cm	LAFF	3.2 × 2.5 cm	None
6	M	62	Temple	10 mm	1.5 × 1.3 cm	LAFF	4 × 4 cm	None
7	F	59	Cheek	10 mm	3 × 2 cm	SCIP	5 × 4 cm	None
8	M	89	Cheek	10 mm	10 × 8 cm	ALT	10 × 8 cm	None
9	F	72	Temple	10 mm	2 × 1 cm	LAFF	4 × 3 cm	None
10	F	58	Cheek	15 mm	3 × 2.5 cm	ALT	8 × 6 cm	None
11	M	85	Chin	20 mm	3 × 2 cm	SCIP	7 × 6 cm	None
12	F	87	Cheek	10 mm	8 × 5 cm	RFFF	8 × 5 cm	None
13	M	70	Lower lip	20 mm	1.5 × 1.2 cm	RFFF	6 × 4 cm	Mild drooling
14	F	49	Cheek	10 mm	2 × 2 cm	ALT	5 × 3 cm	None

RFFF: radial forearm free flap; SCIP: superficial circumflex iliac artery perforator flap; LAFF: lateral arm free flap; PAFF: preauricular free flap; ALT: anterolateral thigh free flap.

## Data Availability

The datasets used and/or analyzed during the current study are available from the corresponding author on reasonable request.

## Abbreviations

SCC	Squamous cell carcinoma
RFFF	Radial forearm free flap
SCIP	Superficial circumflex iliac artery perforator flap
LAFF	Lateral arm free flap
PAFF	Preauricular free flap
ALT	Anterolateral thigh free flap
